# Longitudinal assessment of the impact of prevalent diabetes on hospital admissions and mortality in the general population: a prospective population-based study with 19 years of follow-up

**DOI:** 10.1186/s12889-024-20435-7

**Published:** 2024-10-24

**Authors:** Madeleine Johansson, Anna Åkesson, Peter M. Nilsson, Olle Melander

**Affiliations:** 1 Department of Clinical Sciences, Lund University, Malmö, Sweden; 2 Department of Cardiology, Skåne University Hospital, Malmö, Sweden; 3 Clinical Studies Sweden – Forum South, Skåne University Hospital, Lund, Sweden; 4 Department of Internal Medicine, Skåne University Hospital, Malmö, Sweden

**Keywords:** Diabetes mellitus, Epidemiology, Hospitalization, ICD-10, Mortality, Risk

## Abstract

**Background:**

Hospitalization indicates the presence of severe disease and constitutes a leading cost in health care. We aimed to prospectively assess if prevalent diabetes mellitus contributes to excess all-cause and cause-specific hospital admissions and mortality at the population level.

**Methods:**

We used a Swedish prospective population-based cohort, including 25,642 individuals of whom 4.2% had prevalent diabetes at baseline (mean age 61.2 ± 6.8 years, age range 44.8–73.4 years). We compared the number of hospitalizations and mortality classified according to the main chapters of the 10th revision of the International Classification of Diseases (ICD-10) during follow-up using nationwide inpatient registries, comparing individuals with and without prevalent diabetes, using multivariate adjusted negative binomial regression (incidence rate ratio, IRR) and Cox regression, respectively.

**Results:**

During a median follow-up of 19 years, 18,904 subjects were hospitalized at least once [median 3 (IQR 2–6)] and 6767 (26.4%) individuals died. Overall, subjects with diabetes were hospitalized (IRR 1.83, *p* < 0.001) more often, and had a higher incidence rate of hospital admissions due to endocrine diseases (IRR 14.6, *p* < 0.001), dermatological diseases (IRR 3.7, *p* < 0.001), injuries and poisoning (IRR 2.7, *p* < 0.001), infectious diseases (IRR 2.5, *p* < 0.001), psychiatric diseases (IRR 2.0, *p* < 0.001), but also cardiovascular, hematological, genitourinary, neurologic and respiratory diseases compared with non-diabetic individuals. No difference was observed for hospital admissions due to cancer or musculoskeletal disorders. All-cause mortality was higher (HR 1.77, *p* < 0.001) in individuals with diabetes, with disease-specific mortality being significant only for cardiovascular and endocrine disease-related death.

**Conclusions:**

At the population level, prevalent diabetes increased the hospitalization burden longitudinally due to diseases of most of the ICD-10 main chapters, except for cancer and musculoskeletal disorders. These novel findings challenge the current view on the spectrum of prevalent diabetes-related conditions and may have implications for screening and treatment strategies in diabetes.

**Permission of graphical illustrations:**

Source: Pixabay.com. No permission or acknowledgement is required.

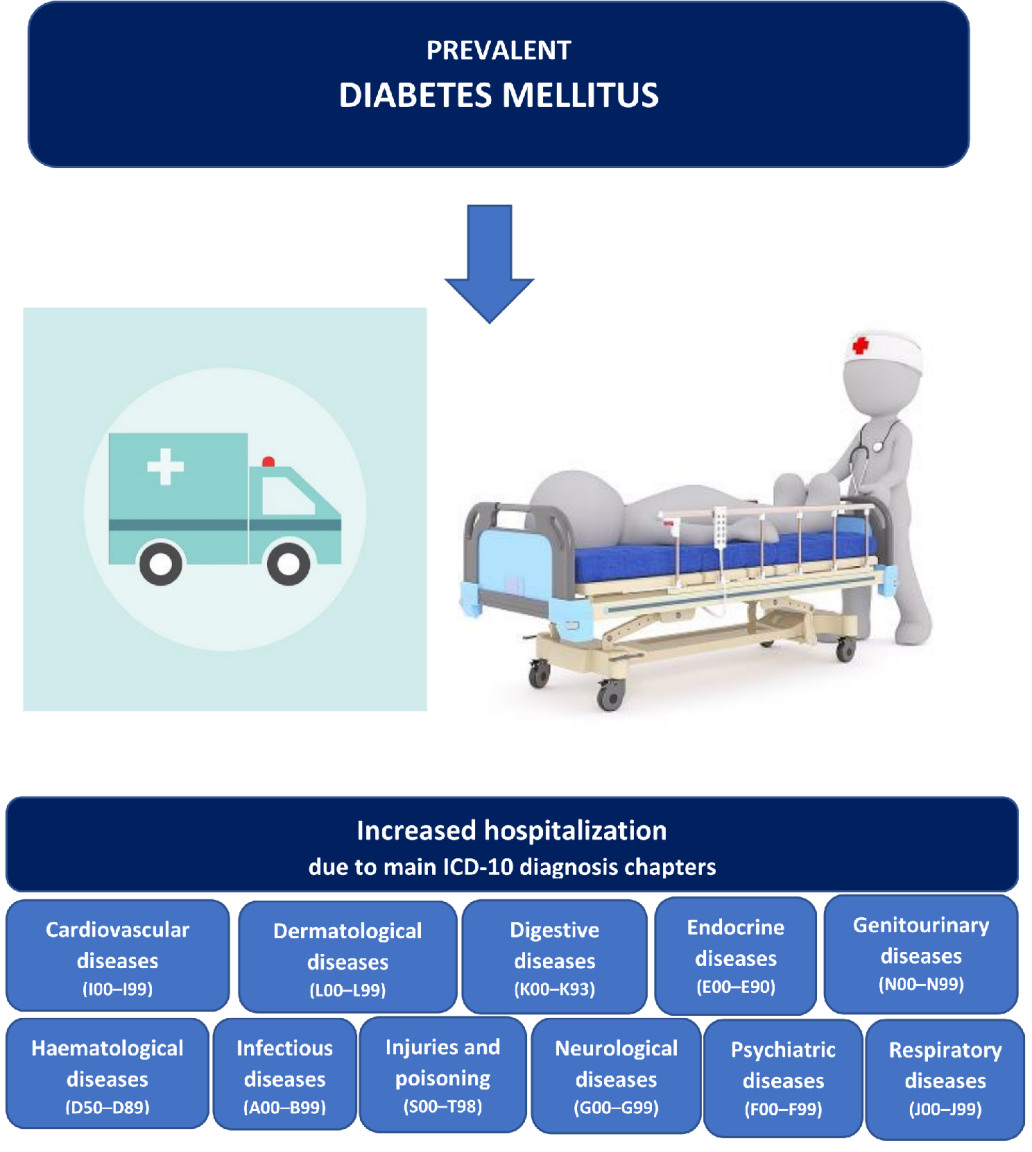

**Supplementary Information:**

The online version contains supplementary material available at 10.1186/s12889-024-20435-7.

## What is already known?

•Diabetes mellitus is a well-known risk factor for increased hospitalization and remains a great public health challenge.

•Considerable opportunities exist to decrease the number of hospitalizations for individuals with diabetes.

**What is the key question?**

Does prevalent diabetes contribute to excess risk of over-all and cause-specific hospitalizations and mortality in the general population?

**What is new?**

•The spectrum of underlying diabetes-associated diseases causing hospitalizations (apart from diabetes itself) has not been studied in a prospective population-based setting before.

•Our longitudinal assessment of the impact of prevalent diabetes in a large Swedish middle-aged population with nearly 19-years of follow-up, found that the concept of what is today regarded as diabetes-related conditions warrants re-evaluation.

**How might this impact clinical practice in the future?**

Our findings underscore which disease categories should be routinely screened for in diabetes patients, both in hospital settings and in outpatient care, motivating earlier clinical work-up based on mild symptoms and structured laboratory, imaging, and functional examinations.

**Supplementary Information:**

The online version contains supplementary material available at 10.1186/s12889-024-20435-7.

## Introduction

Diabetes is one of the most common chronic medical conditions worldwide. The prevalence of diabetes in the US population reached 34.2 million (10.5%) in 2018 [1], and rates are expected to increase further primarily due to an aging population, changing racial/ethnic diversity, as well as changes in obesity rates and lifestyle factors [2].

Steadily rising life-expectancy in large parts of the world has resulted in an increasing proportion of elderly people in society and as a result the need of and costs related to hospitalizations for acute or chronic diseases have increased [3, 4]. The health economic implications related to increased needs of inpatient care are enormous [5].

One of the keys in tackling this public health challenge lies in population-wide primary preventive life-style strategies of non-communicable diseases, as well as more efficient screening and prevention of complications in individuals with established disease [4]. In the context of pre-diabetes, lifestyle intervention has been proven to effectively prevent development of type 2 diabetes [6, 7], and in established type 2 diabetes cardiovascular risk factor control is essential in preventing macro- and microvascular complications, but also mortality [8, 9].

Even though it is known that diabetes increases the risk of hospitalizations [10–14] the spectrum of underlying diabetes-associated diseases causing such hospitalizations (apart from diabetes itself) have, to the best of our knowledge, not been studied in a prospective population-based setting before. To address this issue, we aimed to longitudinally assess the impact of prevalent diabetes on the risk of all-cause and disease-specific hospitalizations and mortality assessed according to the main diagnosis chapters of the International Classification of Diseases version 10 (ICD-10) system during 19-years of follow-up in the Swedish general population.

## Methods

### Study population and design

The Malmö Diet and Cancer study (MDCS) is a large prospective population-based study initiated in the 1990s [15]. Participants born between 1923 and 1950 living in the city of Malmö, Sweden, were eligible for participation. A total of 74,138 subjects were invited to participate, of whom 30,447 attended the baseline examination between 1991 and 1996. Details of the MDCS have been described elsewhere [16, 17]. After exclusion of 4,805 individuals due to missing data from the questionnaire, clinical exam variables, or serum sample measurement of apolipoprotein B (ApoB) and apolipoprotein A1 (ApoA1) concentrations, a total of 25,642 individuals with complete data on covariates were included in the final analysis. Data on hospitalizations and mortality was acquired from the Swedish National Inpatient Register [18] and Swedish National Cause of Death Register [19], with virtually complete coverage of the total Swedish population. All participants were followed until emigration from Sweden, death or the end of follow-up on December 31, 2014.

### Definition of baseline variables

The presence of diabetes mellitus at baseline was defined as one or more of the following criteria: a self-reported physician´s diagnosis of diabetes or use of anti-diabetic medication based on the MDCS questionnaire; a fasting plasma glucose measurements of ≥ 126 mg/dL (≥ 7.0 mmol/L) taken at the baseline examination; or a diagnosis of diabetes in any of the local or national diabetes registries prior to study entry: The Malmo HbA_1c_ register, The Swedish National Diabetes Register, The Regional Diabetes 2000 Register of the county council (Region Skåne), The Swedish National Patient Register covering all hospital discharges and hospital based outpatient care, The Swedish National Prescribed Drug Register (prescription of anti-diabetic medication), and The Swedish Cause-of-Death Register [20–24]. The type of diabetes mellitus was not specified in all registries.

Serum concentrations of ApoA1 and ApoB were measured by Quest Diagnostics (San Juan Capistraon, CA), blinded to case-control status, using an immunonephelometric assay run on the Siemens BNII (Siemens, Newark, DE). The inter-assay variability was < 4.0% for both ApoA1 and ApoB. Blood pressure (BP) was measured twice in the supine position after 5 min of rest, using a standard mercury sphygmomanometer on the right arm. The average of these two values was recorded as the actual BP level. Smoking status was self-reported and coded as 0 = never, 1 = former or 2 =current (i.e. any smoking within the past year) as a categorical variable. The anthropometric and body composition examination was conducted without prior fasting. Weight was measured to the nearest 0.1 kg using a balance-beam scale with the subjects wearing light clothing and no shoes. Standing height was measured with a fixed stadiometer calibrated in centimeters. Body mass index (BMI) was calculated as weight in kilograms divided by height in meters squared (kg/m2). Education level was divided into four categories according to highest level of education: ≤8 years, 9–10 years, 11–12 years, and college education/university degree.

### Classification of hospitalizations and mortality according to ICD-10

Hospitalizations were defined as inpatient hospital admissions > 24 h. We classified the causes of hospitalizations and mortality based on the main ICD-10 discharge diagnosis of the National Inpatient Register and National Cause of Death Register, respectively. The following main ICD-10 chapters and codes were used to define disease groups in the analysis:

- A00–B99, Certain infectious and parasitic diseases, “infectious diseases”.
- C00–D48, Neoplasms, hereafter termed “cancer”.
- D50–D89, Hematological diseases and certain disorders involving the immune mechanism, hereafter termed “hematological diseases”.
- E00–E90, Endocrine, nutritional, and metabolic diseases, hereafter termed “endocrine diseases”.
- F00–F99, Mental and behavioral disorders, hereafter termed “psychiatric diseases”.
- G00–G99, Neurological diseases.
- I00–I99, Circulatory diseases, hereafter termed “cardiovascular diseases”.
- J00–J99, Respiratory diseases)
- K00–K93, Digestive diseases.
- L00–L99, Skin and subcutaneous tissue diseases, hereafter termed “dermatological diseases”.
- M00–M99, Musculoskeletal and connective tissue diseases, hereafter termed “musculoskeletal diseases”.
- N00–N99, Genitourinary diseases.
- R00–R99, Symptoms, signs and abnormal clinical and laboratory findings, not elsewhere classified, hereafter termed “unspecified symptoms”.
- S00–T98, Injury, poisoning, and certain other consequences of external causes, hereafter termed “injuries and poisoning”.

The following two ICD-10 chapters were excluded due to few cases: 4 and 35 hospitalizations, respectively, and correspondingly 0 and 3 deaths, respectively:

- O00–O99, Pregnancy, childbirth, and the puerperium, hereafter termed “pregnancy-related complications”.
- Q00–Q99, Congenital malformations, deformations, and chromosomal abnormalities, hereafter termed “congenital diseases”.

### Exposure and outcome measures

The exposure was prevalent diabetes at the baseline exam. The primary outcome was the number of hospitalizations in each subject of the total population during 19-years of follow-up, including total number of hospitalizations and numbers of disease-specific hospitalizations attributed to each ICD-10 chapter. The secondary outcome was all-cause and cause-specific mortality during follow-up.

### Ethical approval

The MDCS was approved by the ethics committee at Lund University (LU 51–90), and all participants provided written informed consent. The study was conducted in compliance with the Declaration of Helsinki and its later amendments.

### Statistical analyses

Continuous data are shown as mean ± standard deviation if not otherwise specified, whereas frequencies are used to describe categorical data. Continuous variables were compared using Student’s t-test and proportions were compared using Pearson’s Chi-square test.

In the primary outcome analysis, negative binomial regression was used to analyze the relationship between baseline diabetes status and number of hospitalizations (all-cause and ICD-10 disease-specific) with reported incidence rate ratio (IRR), 95% confidence interval (95%CI), and log-transformed follow-up time, considering that the primary outcome had a skewed distribution with a large proportion of participants never being hospitalized and a proportion with increasing numbers of hospitalizations during the follow-up time. We did not report the IRR for hospitalizations with less than 10 cases.

In the secondary outcome analysis, Cox Proportional Hazards Model was used to analyze the relationship between diabetes status and all-cause and cause-specific mortality with reported hazard ratio (HR) and 95% CI. We did not report the HR for cause-specific deaths with less than 10 cases.

Both the primary and secondary outcome analyses were adjusted for two different models. *Model 1* was adjusted for age and sex. *Model 2* was adjusted for age, sex, serum concentrations of ApoA1 and ApoB, current smoking, BMI, antihypertensive treatment, systolic BP, prevalent CVD, and education level. To counteract the problem of multiple comparisons, Bonferroni-corrected P-value was calculated in *Model 2*. To reduce the risk of confounding, we performed a sub-analysis excluding subjects with prevalent diseases in each respective ICD-10 chapter.

All statistical analyses were performed in IBM SPSS Statistics 27 (IBM Corporation, Armonk, NY, USA).

## Results

The original study sample (*n* = 30,447) is presented in the Supplementary Table S1. Out of the total study population of 25,642 subjects, 1065 individuals (4.2%) had prevalent diabetes (mean age 61.2 ± 6.8 years, age range 44.8–73.4 years) at the MDCS baseline examination 1991–1996. During a median follow-up period of 18.9 ± 4.4 years, overall, 90.6% (*n* = 965) subjects with diabetes versus 73.0% (*n* = 17,939) without diabetes were hospitalized, while 51.1% (*n* = 544) subjects with diabetes died compared with 25.3% (*n* = 6,223) without diabetes.

Baseline study characteristics are shown in Table 1. Individuals with diabetes were older, more likely to be men and treated with anti-hypertensive medications. They also had higher BMI, ApoB, blood pressure, lower ApoA1 levels, and fewer had a college or university degree.

**Table 1:** Baseline characteristics of the study population (*n* = 25,642)

	Prevalent diabetes (*n* = 1,065)	No diabetes (*n* = 24,577)	*P*-value
**Age (years ± SD)** **(Age range)**	61.2 ± 6.8 (44.8–73.4)	58.0 ± 7.7 (44.5–73.6)	**< 0.001**
**Sex (%**,** women)**	47.2	61.4	**< 0.001**
**Follow-up time (years ± SD)**	15.7 ± 5.5	17.8 ± 4.4	**< 0.001**
**BMI (kg/m**^**2**^ **± SD)**	28.1 ± 4.6	25.6 ± 3.9	**< 0.001**
**Systolic BP (mmHg ± SD)**	150.0 ± 19.8	140.9 ± 20.0	**< 0.001**
**Anti-hypertensive treatment (%)**	44.3	17.2	**< 0.001**
**Current smoking (%)**	24.5	28.0	**0.01**
**ApoA1 (mg/dL ± SD)**	147.9 ± 28.0	157.1 ± 28.0	**< 0.001**
**ApoB (mg/dL ± SD)**	113.8 ± 28.15	106.6 ± 25.8	**< 0.001**
**Educational level (%)**			**< 0.001**
**≤8 years** **9–10 years** **11–12 years** **College/university degree**	50.2 23.7 8.5 17.1	41.5 26.1 8.1 23.2	
**Prevalent diseases at baseline according to ICD-10 chapters (%)**			
**Infectious diseases (A00–B99)** **Cancers (C00–D48)** **Hematological diseases (D50–D89)** **Endocrine diseases (E00–E90)** **Psychiatric diseases (F00–F99)** **Neurological diseases (G00–G99)** **Cardiovascular diseases (I00–I99)** **Respiratory diseases (J00–J99)** **Digestive diseases (K00–K93)** **Dermatological diseases (L00–L99)** **Musculoskeletal diseases (M00–M99)** **Genitourinary diseases (N00–N99)** **Pregnancy-related complications (O00–O99)** **Congenital diseases (Q00–Q99)** **Unclassified symptoms (R00–R99)** **Injuries and poisoning (S00–T98)**	4.2 13.4 0.6 3.7 5.4 7.9 24.8 8.7 25.0 2.9 12.3 18.4 4.5 0.8 15.9 14.1	3.3 13.5 0.4 2.0 3.1 4.1 11.1 6.0 16.1 1.3 7.9 14.1 11.7 0.7 11.7 10.8	0.09 0.09 0.34 **< 0.001** **< 0.001** **< 0.001** **< 0.001** **< 0.001** **< 0.001** **< 0.001** **< 0.001** **< 0.001** **< 0.001** 0.79 **< 0.001** **0.001**

*Abbreviations*: ApoA1, apolipoprotein A1; ApoB, apolipoprotein B; BMI, body mass index; BP, blood pressure; CVD, cardiovascular disease; SD, standard deviation

### Hospitalizations during 19 years of follow-up in subjects with prevalent diabetes

The total and disease-specific number of hospitalizations and incidence rates for subjects with prevalent diabetes is shown in Table 2. A total of 18,904 subjects were hospitalized at least once for any cause [median 3 (interquartile range, IQR 2–6)]. Subjects with diabetes had an overall increased risk of hospitalizations (IRR: 1.83, 95%CI 1.71–1.96, Bonferroni-adjusted p = < 0.001, *Model 2*, Table 2). In the fully adjusted *Model 2*, diabetes remained significantly associated with increased number of hospitalizations for all disease groups, except for cancer, musculoskeletal diseases (Table 2). The number of hospital admissions due to specific cancer types in subjects with diabetes is shown in Supplementary Table S2.

**Table 2:** Incidence rate of hospital admissions according to the main ICD-10 chapters. Comparison between individuals with or without prevalent diabetes (*n* = 25,642)

Hospitalizations according to main ICD-10 chapters (ICD-10 codes)			Model 1		Model 2		
	Number of individuals in the total population with ≥ 1 hospitalization	Median (IQR)	IRR (95% CI)	*P*-value	IRR (95% CI)	*P*-value	Bonferroni corrected *p*-value
**All-cause hospitalizations**	18,904	3 (2–6)	2.14 (2.00–2.29)	**< 0.001**	1.83 (1.71–1.96)	**< 0.001**	**< 0.001**
**Infectious diseases** **(A00–B99)**	1,645	1 (1–1)	3.07 (2.65– 3.55)	**< 0.001**	2.54 (2.18–2.95)	**< 0.001**	**< 0.001**
**Cancers** **(C00–D48)**	6,561	2 (1–3)	0.94 (0.85– 1.04)	0.20	0.91 (0.82–1.01)	0.08	NS
**Hematological diseases** **(D50–D89)**	486	1 (1–1)	1.86 (1.38– 2.51)	**< 0.001**	1.64 (1.21–2.22)	**0.002**	**0.03**
**Endocrine diseases** **(E00–E90)**	1,323	1 (1–2)	16.66 (14.95–18.57)	**< 0.001**	14.56 (13.02–16.29)	**< 0.001**	**< 0.001**
**Psychiatric diseases** **(F00–F99)**	630	1 (1–2)	2.09 (1.63– 2.68)	**< 0.001**	2.03 (1.57–2.61)	**< 0.001**	**< 0.001**
**Neurological diseases** **(G00–G99)**	1,680	1 (1–1)	1.96 (1.67– 2.31)	**< 0.001**	1.78 (1.51–2.10)	**< 0.001**	**< 0.001**
**Cardiovascular diseases** **(I00–I99)**	8,690	2 (1–4)	2.39 (2.21– 2.59)	**< 0.001**	1.85 (1.71–2.00)	**< 0.001**	**< 0.001**
**Respiratory diseases** **(J00–J99)**	3,508	1 (1–2)	1.63 (1.45– 1.82)	**< 0.001**	1.69 (1.50–1.90)	**< 0.001**	**< 0.001**
**Digestive diseases** **(K00–K93)**	5,274	1 (1–2)	1.49 (1.34– 1.66)	**< 0.001**	1.32 (1.18–1.47)	**< 0.001**	**< 0.001**
**Dermatological diseases** **(L00–L99)**	257	1 (1–1)	4.49 (3.32– 6.08)	**< 0.001**	3.68 (2.69–5.04)	**< 0.001**	**< 0.001**
**Musculoskeletal diseases** **(M00–M99)**	4,502	1 (1–2)	1.32 (1.17– 1.49)	**< 0.001**	1.13 (1.00–1.28)	0.55	NS
**Genitourinary diseases** **(N00–N99)**	4,147	1 (1–2)	2.13 (1.91– 2.37)	**< 0.001**	1.82 (1.62–2.03)	**< 0.001**	**< 0.001**
**Unclassified symptoms**,** (R00–R99)**	3,425	1 (1–2)	2.01 (1.78– 2.28)	**< 0.001**	1.72 (1.52–1.96)	**< 0.001**	**< 0.001**
**Injuries and poisoning** **(S00–T98)**	661	1 (1–1)	2.70 (2.13– 3.43)	**< 0.001**	2.71 (2.13–3.46)	**< 0.001**	**< 0.001**

*Abbreviations*: CI, confidence interval; ICD-10, 10th revision of the International Classification of Diseases; IQR, interquartile ranges; IRR, incidence rate ratio; NS, not significant

Negative binomial regression analysis of number of hospitalizations in patients with prevalent diabetes mellitus compared with non-diabetic, stratified according to the main ICD-10 chapters with reported incidence rate ratio

*Model 1*: adjusted for age and sex

*Model 2*: adjusted for age, sex, apolipoprotein A1, apolipoprotein B, body mass index, current smoking, antihypertensive treatment, and systolic blood pressure

As expected, individuals with prevalent diabetes had the highest incidence rate of hospitalizations due to endocrine diseases (IRR: 14.56, 95% CI 13.02–16.29, *p* < 0.001, Table 2, *Model 2*), whereas around a double incidence rate of hospitalizations due to infectious, dermatological, and psychiatric diseases was observed, as well as for hospitalizations due to injuries and poisoning. For hospitalizations due to digestive, hematological, respiratory, neurological, genitourinary, and cardiovascular diseases, as well as for unspecified symptoms and abnormal clinical and laboratory findings, the incidence rate was higher for subjects with prevalent diabetes (IRR 1.32–1.82, Table 2, *Model 2*).

### Mortality during 19 years of follow-up in subjects with prevalent diabetes

The total and disease-specific number of deaths and mortality risk for subjects with prevalent diabetes is shown in Table 3. Overall, subjects with diabetes had higher risk of all-cause mortality (HR: 1.77, 95%CI 1.62–1.93, Bonferroni-corrected *p* < 0.001, Table 3, *Model 2*), and disease-specific mortality due to endocrine diseases, infectious, and cardiovascular diseases, as well as death caused by injuries and poisoning (Table 3, *Model 1*). After full Bonferroni-adjustment, prevalent diabetes remained significantly related to death due to two of these four disease groups (Table 3, *Model 2*).

**Table 3:** Mortality according to the main ICD-10 chapters. Comparison between individuals with and without prevalent diabetes (*n* = 25,642)

Deaths according to main ICD-10 chapters (ICD-10 codes)		Model 1		Model 2		
	Number of deaths in total population (%)	HR (95% CI)	*P*-value	HR (95% CI)	*P*-value	Bonferroni corrected *p*-value
**All-cause deaths**	6767 (26.4)	1.92 (1.76–2.09)	**< 0.001**	1.77 (1.62–1.93)	**< 0.001**	**< 0.001**
**Infectious diseases** **(A00–B99)**	99 (0.4)	2.57 (1.33–4.94)	**0.005**	2.14 (1.10–4.17)	**0.03**	NS
**Cancers** **(C00–D48)**	2,678 (10.4)	1.15 (0.96–1.37)	0.13	1.12 (0.94–1.34)	0.22	NS
**Haematological diseases** **(D50–D89)**	12 (0.00)	4.61 (1.00–21.29)	0.05	2.72 (0.55–13.54)	0.22	NS
**Endocrine diseases** **(E00–E90)**	151 (0.6)	21.15 (15.33–29.19)	**< 0.001**	16.22 (11.55–22.80)	**< 0.001**	**< 0.001**
**Psychiatric diseases** **(F00–F99)**	237 (0.9)	1.30 (0.73–2.33)	0.37	1.33 (0.74–2.39)	0.35	NS
**Neurological diseases** **(G00–G99)**	272 (1.1)	1.07 (0.60–1.91)	0.82	1.15 (0.64–2.05)	0.65	NS
**Cardiovascular diseases** **(I00–I99)**	2,223 (8.7)	2.51 (2.19–2.87)	**< 0.001**	2.08 (1.81–2.39)	**< 0.001**	**< 0.001**
**Respiratory diseases** **(J00–J99)**	406 (1.6)	1.25 (0.80–1.93)	0.33	1.42 (0.91–2.22)	0.12	NS
**Digestive diseases** **(K00–K93)**	187 (0.7)	1.65 (0.94–2.89)	0.08	1.44 (0.81–2.56)	0.21	NS
**Dermatological diseases** **(L00–L99)**	Only 9 deaths					
**Musculoskeletal diseases** **(M00–M99)**	28 (0.1)	0.87 (0.12–6.43)	0.89	1.04 (0.14–7.81)	0.97	NS
**Genitourinary diseases** **(N00–N99)**	56 (0.2)	2.18 (0.87–5.49)	0.10	2.06 (0.81–5.24)	0.13	NS
**Unclassified symptoms**,** (R00–R99)**	170 (0.7)	1.64 (0.89–3.03)	0.11	1.54 (0.83–2.87)	0.17	NS
**Injuries and poisoning(S00–T98)**	215 (0.8)	1.72 (1.03–2.87)	**0.04**	1.75 (1.04–2.94)	**0.04**	NS

*Abbreviations*: CI, confidence interval; HR, hazard ratio; ICD-10, 10th revision of the International Classification of Diseases; NS, not significant

Cox regression analysis of number of deaths in patients with prevalent diabetes mellitus compared with non-diabetic stratified according to the main ICD-10 chapters with reported hazard ratio

*Model 1*: adjusted for age and sex

*Model 2*: adjusted for age, sex, apolipoprotein A1, apolipoprotein B, current smoking, body mass index, antihypertensive treatment, and systolic blood pressure

Competing risk analysis of the effect of prevalent diabetes on cardiovascular and cancer mortality, showed no effect of diabetes on cancer mortality in the fully-adjusted *Model 2*: SHR: 0.99, 95% CI 0.83–1.19, *p* = 0.95.

To reduce the risk of confounding, we excluded individuals with diseases in each ICD-10 chapter, respectively. All results for hospitalizations (Supplementary Table S3, *Model 2*) and mortality remained significant (Supplementary Table S4, *Model 2*), except for hospitalizations due to digestive diseases which did not differ.

We performed a sensitivity analysis excluding individuals who developed diabetes at any time during the follow-up (i.e., incident diabetes) for hospital admissions (Supplementary Table S5, *Model 2*) and mortality (Supplementary Table S6, *Model 2*). The results remained virtually unchanged (*Model 2* after Bonferroni correction).

## Discussion

In this population-based cohort study followed prospectively for 19 years, with virtually complete nationwide coverage of hospitalizations and deaths, we observed that individuals with prevalent diabetes at baseline had 1.83 times higher incidence rate ratio of hospitalizations than individuals without diabetes. Notably, hospitalizations were increased for diagnoses of most ICD-10 chapters, indicating that diabetes increases the risk of hospital admissions, i.e. severe disease, in virtually all organs and disease groups, except for musculoskeletal diseases and cancer (mainly prostate, breast, and colorectal cancer). The risk of all-cause mortality nearly doubled in patients with prevalent diabetes, with cardiovascular diseases being the main contributor to the excess mortality risk. Our observations regarding the independent relationship between prevalent diabetes and hospitalizations due to hematological, psychiatric, neurological, dermatological, and unclassified diseases, as well as due to injuries and poisoning, represent largely novel findings.

### Description of the Swedish health care system and prevalent diabetes care in Sweden

The Swedish healthcare system is divided into three administrative levels which are all governed by democratically elected politicians [25]. The system is largely tax-funded, providing all citizens with universal healthcare. In 1943, the “National Diabetes Association in Sweden” for patients was launched, resulting in significant progress of diabetes care in Sweden. Information from the St. Vincent declaration in 1989 contributed to the establishment of the first national Swedish guidelines for diabetes care in 1996, and the launching of the Swedish National Diabetes Register [26]. The registry provides real-life data on the current quality of care and trends over time for diabetes outcome measures, which can be used as a tool for monitoring and quality control of Swedish diabetes care. The information reported to the registry comprises clinical data, risk factor control and the occurrence of comorbidities in people with diabetes mellitus. All individuals provide written informed consent, and general practitioners or registered nurses at primary health care centers are responsible for reporting clinical data to the registry at least annually using online forms. To follow an internationally accepted and unbiased way of classification, we grouped the main hospital discharge diagnoses according to ICD-chapters, while still maintaining subgroups large enough to handle statistically and avoid spurious associations due to multiple comparisons by multiple subgroups.

### Hospital admissions due to cardiovascular diseases (ICD-10 codes I00–I99)

We observed that prevalent diabetes robustly increased the incidence rate ratio of hospital admissions due to cardiovascular diseases by 1.85 during follow-up after adjustment for baseline cardiovascular risk factors. The powerful and well-established link between diabetes and cardiovascular disease [27, 28] was further reinforced by our finding that the excess mortality risk in individuals with diabetes was strongly driven by deaths due to cardiovascular disease, underscoring the need for aggressive pharmacological and non-pharmacological preventive actions in patients with diabetes [29].

### Hospital admissions due to psychiatric diseases (ICD-10 codes F00–F99)

Previous studies have demonstrated that psychiatric disease may increase the risk of new-onset diabetes [30, 31] and associates with prevalent diabetes [32]. Our study is the first to demonstrate that prevalent diabetes may be prospectively related to a two-fold increased risk of incident hospital admissions due to psychiatric diseases, indicating that psychiatric disease ought to be considered as a novel diabetes-related condition. The strong link between diabetes and hospitalization for psychiatric disease during follow-up suggests that diabetes increases the risk not only for mild forms of mood disorders and cognitive impairment, but also for severe psychiatric disease. Our results highlight the importance of screening for e.g. mood disorders and cognitive impairment in the regular management of diabetes [33]. Further studies are needed to fully establish the relationship between diabetes, cognitive decline, and dementia.

### Hospital admissions due to dermatological diseases (ICD-10 codes L00–L99)

Beyond endocrinological diseases, the largest incidence rate of hospital admissions in subjects with prevalent diabetes resulted from dermatological diseases, accounting for a 3.7 times higher incidence in diabetes compared with subject without diabetes. Previous studies have highlighted that skin disorders are oftentimes neglected and underdiagnosed among patients with diabetes [34, 35]. Our study is the first to prospectively link prevalent diabetes to increased incidence of hospital admissions resulting from dermatological diseases. We hypothesize that since the most common diabetes complications of skin and subcutaneous tissue are caused by arterial insufficiency and/or neuropathy in the proximal leg and foot, and coded as diabetes complications, thus falling under the ICD-chapter of “endocrine diseases”, prevalent diabetes can be considered a risk marker for future hospitalizations resulting from primary dermatological diseases, thereby adding further complexity to the screening of dermal and cutaneous problems in diabetes.

### Hospital admissions due to infectious diseases (ICD-10 codes A00–B99)

In the present study we also found that subjects with prevalent diabetes had 2.5 times increased incidence of hospitalization due to infectious diseases compared with individuals without diabetes after multivariate adjustment. Retrospective studies have shown that diabetes confers an increased risk of developing and dying from an infectious disease, in particular skin and soft tissue infections, genitourinary, gastrointestinal, and respiratory infections [36, 37].

### Hospital admissions due to neurological diseases (ICD-10 codes G00–G99)

Similarly, we noted an 1.78 increased incidence rate ratio of hospital admissions due to neurological diseases in diabetes than among non-diabetics. However, this increase is unlikely to stem from diseases such as diabetic neuropathy or stroke, since these diseases are coded under the ICD-10 chapter for endocrine diseases (E11.4) and cardiovascular diseases (I63.9), respectively. Consequently, our data suggest that prevalent diabetes is in fact a risk marker for developing neurological diseases that are not typically encompassed within the category ‘neuropathy-complications’ or dependent on stroke. These findings are largely novel. To date, one prospective registry-based Danish study has demonstrated a link between neurological diseases in general and diabetes mellitus, however, they lacked adjustments for important covariates such as BMI, smoking, cholesterol, etc [38]. , while another one investigated the link with Parkinson’s disease specifically [39].

### Hospital admissions due to hematological diseases (ICD-10 codes D50–D89)

In our study, we found a 1.60 increased incidence rate ratio of hospital admissions due to hematological diseases in subjects with prevalent diabetes. We hypothesize that it most likely results from hospitalizations due to generalized anemia. The presence of anemia in general increases the risk of diabetic complications, including peripheral neuropathy and cardiovascular disease [40–42]. Thus, a heightened awareness of the potential impact of anemia in the diabetes population should be further promoted with identification of patients at high risk of anemia.

### Hospital admissions due to unclassified symptoms (ICD-10 codes R00–R99)

In the present study we found that unclassified symptoms (R00–R99), constituted a very common cause of hospital admissions in subjects with diabetes, with 1.70 times higher incidence rate ratio in diabetes and over 3400 admissions compared with non-diabetic subjects. Considering that various complaints such as chest pain or abdominal pain often cause hospitalization, it may be hypothesized that it is of particular importance not to dismiss or overlook these complaints in individuals with diabetes, why these aspects warrant further investigations.

### Public importance of preventing hospitalizations in prevalent diabetes

The multifactorial nature of diabetes requires a comprehensive multifactorial management approach. A large proportion of hospitalizations for patients with diabetes may be prevented. In fact, it is estimated that one-fifth of hospitalizations in individuals aged ≥ 65 years with diabetes may be prevented [43]. The direct costs of hospitalizations are 4-fold higher in older individuals (≥ 65 years) with prevalent diabetes [44].

Since treatment of diabetes is strongly dependent on outpatient care, hospitalizations due to uncontrolled diabetes or complications may largely mirror the primary health care structure and quality offered outside of the hospital [45]. Therefore, it is plausible to assume that timely and appropriate outpatient care may prevent hospitalizations.

Considerable opportunities exist to decrease the number of hospitalizations for patients with diabetes, for example by addressing risk factors that precipitate hospitalizations and avoidance of hypoglycemia, but also avoidance of drug-related hospitalizations, such as polypharmacy, and adverse effects due to antihypertensive medications.

### Strengths and limitations of this study

The strengths of our study include the large population-based cohort with little loss to follow-up, and that data on hospitalizations and mortality was retrieved from national registries fully covering the Swedish population, which makes the data reliable and robust. Moreover, individuals with prevalent diseases in the respective ICD-10 chapters were excluded to reduce the risk of confounding. To the best of our knowledge the full spectrum of underlying diabetes-associated diseases responsible for hospitalizations have not been explored in a prospective population-based setting like this before. It is important to underline that the present study is an epidemiological evaluation focusing on identifying prevalent diabetes-related conditions regardless of whether the disease was causally related or only a parallel phenomenon.

As far as limitations are concerned, there was a substantial number of individuals with missing data for all variables in the initial dataset (*n* = 30,447). Excluded individuals were younger, more likely to be men, and had a higher mortality rate, e.g., the excluded individuals were likely sicker than those included. Furthermore, the diseases were classified according to the broad classification of ICD-10 chapters. Consequently, the representation of certain complaints can be misleading, for example urinary infections are found under the ICD-10 chapter of “Diseases of the genitourinary system”, and not under the chapter “Certain infectious and parasitic diseases”, as is pneumonia, which is found under “Disease of respiratory system” and not under “Certain infectious and parasitic diseases”. Moreover, we focused on the main discharge diagnoses since these diagnoses have been validated by the Swedish National Inpatient Register [23], and indicate disease severity and are thus of clinical relevance.

To reduce the risk of confounding, we excluded individuals with prevalent diseases in the respective ICD-10 chapters, nevertheless, the risk of residual confounding may remain. Furthermore, very few deaths and hospitalizations due to pregnancy-related complications or congenital and chromosomal abnormalities were reported in the study population, which may be partly explained by the higher mean age of individuals included in the study, consequently these two ICD-10 chapters were not reported in the present study. Also, the type of diabetes was not specified in the various diabetes registries. Finally, as with every chronic disease condition associated with frequent health care visits, a component of increased detection bias cannot be ruled out. Further studies in other ethnic populations and age-groups are needed to investigate the generalizability of our results.

### Meaning of study findings

Overall, our study indicates that the concept of what is today regarded as diabetes-related conditions might need to be re-evaluated, underscoring which diseases categories should be routinely screened for in subjects with diabetes, both in hospital settings and outpatient care. Our findings might motivate earlier clinical work-up based on mild symptoms and possibly structured laboratory, imaging, and functional examinations, focusing on detection and prevention of the various diseases that lead to excess hospitalizations in subjects with diabetes.

## Conclusions

This population-based study demonstrated that subjects with prevalent diabetes had 1.83 times higher incidence of hospital admissions in general during 19 years of follow up. The incidence rate ratio of disease-specific hospital admissions was between 1.30 and 2.70 times higher compared with non-diabetic subjects. Beyond higher incidence rates for hospitalizations due to endocrine diseases, most hospital admissions were related to dermatological diseases (3.7-fold higher), injuries and poisoning (2.7-fold higher), infectious diseases (2.5-fold higher) psychiatric diseases (2-fold higher incidence). No difference was observed for hospital admissions due to cancer or musculoskeletal disorders. All-cause mortality was 77% higher in individuals with prevalent diabetes, with disease-specific mortality being significant only for cardiovascular and endocrine disease-related death. Our findings challenge the current view on the limited spectrum of diabetes-related clinical conditions and may have implications for screening and treatment strategies in diabetes.

## Electronic supplementary material

Below is the link to the electronic supplementary material.

Supplementary Material 1

## Data Availability

The datasets analyzed during the current study are available from the corresponding author on reasonable request.

## Abbreviations

ApoA1: Apolipoprotein A1
ApoB: Apolipoprotein B
BP: Blood pressure
ICD-10: International Classification of Diseases version 10
MDCS: The Malmö Diet and Cancer Study
