# Supplementary material for: Longitudinal assessment of the impact of prevalent diabetes on hospital admissions and mortality in the general population: a prospective population-based study with 19 years of follow-up
Source: BMC Public Health. 2024 Oct 24;24:2948. doi: 10.1186/s12889-024-20435-7 (PMC11515336; doi:10.1186/s12889-024-20435-7)
Supplement: Supplementary file 1 — Supplementary Material 1 [file 12889_2024_20435_MOESM1_ESM.docx]

**SUPPLEMENT**

**Table S1. Original study sample (n=30,447) prior to exclusion.**

|  | **Excluded individuals (*n*=4,805)** | **Included individuals (*n*=25,642)** | **p-value** |
| --- | --- | --- | --- |
| **Age (years ± SD)** | 57.3 ± 7.0 | 58.2 ± 7.7 | **<0.001** |
| **Sex (%, men)** | 43.3 | 39.2 | **<0.001** |
| **All-cause mortality (%)** | 32.1 | 26.4 | **<0.001** |

**Table S2. Number of main first admissions to hospital according to the main ICD-10 codes related to prevalent diabetes mellitus.**

| **The most common main ICD-10 diagnosis during first admission to hospital** | **Number of individuals with**  **≥ 1 first disease-specific hospital admission**  **ICD-9 codes** | **Number of individuals with**  **≥ 1 first disease-specific hospital admission**  **ICD-10 codes** |
| --- | --- | --- |
| **Infectious diseases**  **(A00–B99)** | **249** | |
| Sepsis, Gram-negative organisms |  | 37 |
| Erysipelas |  | 35 |
| Sepsis, unspecified organism |  | 21 |
| **Cancers**  **(C00–D48)** | **3,556** | |
| Breast cancer | 146 | 441 |
| Prostate cancer | 87 | 332 |
| Colorectal cancer | 65 | 256 |
| Bladder cancer |  | 136 |
| **Hematological diseases**  **(D50–D89)** | **89** | |
| Iron deficiency anemia |  | 38 |
| Anemia, unspecified |  | 19 |
| **Endocrine diseases**  **(E00–E90)** | **419** | |
| Nontoxic multinodular goiter |  | 32 |
| Primary hyperparathyroidism |  | 29 |
| Diabetes mellitus | 107 | 24 |
| **Psychiatric diseases**  **(F00–F99)** | **158** | |
| Unspecified dementia |  | 17 |
| Delirium, unspecified |  | 10 |
| Alcohol dependence syndrome | 29 |  |
| **Neurological diseases**  **(G00–G99)** | **465** | |
| Transient cerebral ischemic attack |  | 107 |
| Alzheimer's disease with late onset |  | 27 |
| Parkinson's disease |  | 25 |
| **Cardiovascular diseases**  **(I00–I99)** | **4,686** | |
| Acute myocardial infarction | 191 | 637 |
| Angina pectoris | 188 | 463 |
| Atrial fibrillation | 100 | 550 |
| Cerebral infarction | 69 | 292 |
| **Respiratory diseases**  **(J00–J99)** | **1,145** | |
| Bacterial pneumonia | 44 | 195 |
| Unspecified pneumonia | 29 | 117 |
| **Digestive diseases**  **(K00–K93)** | **2,553** | |
| Cholelithiasis | 139 | 229 |
| Inguinal hernia | 85 | 132 |
| Diverticular disease of large intestine | 77 | 126 |
| **Dermatological diseases**  **(L00–L99)** | **42** | |
| Cutaneous abscess, furuncle and carbuncle of limb |  | 3 |
| Pressure ulcer |  | 3 |
| **Musculoskeletal diseases**  **(M00–M99)** | **2,251** | |
| Osteoarthritis (hip, knee, carpometacarpal joint) | 150 | 835 |
| Spinal stenosis |  | 84 |
| Rheumatoid arthritis | 50 | 33 |
| **Genitourinary diseases**  **(N00–N99)** | **2,026** | |
| Benign prostatic hyperplasia | 87 | 238 |
| Prolapsed bladder | 99 | 144 |
| Stress incontinence | 97 | 111 |
| Urinary tract infection | 17 | 86 |
| **Pregnancy-related complications (O00–O99)** | **4** | |
| **Congenital diseases**  **(Q00–Q99)** | **7** | |
| **Unclassified symptoms, abnormal clinical and laboratory findings (R00–R99)** | **769** | |
| Syncope and collapse | 52 | 238 |
| Chest pain, unspecified | 26 | 90 |
| Dizziness | 6 | 49 |
| **Injuries and poisoning**  **(S00–T98)** | **485** | |
| Fracture of neck of femur | 64 |  |
| Concussion | 37 |  |
| Fracture of radius and ulna | 29 |  |

**Table S3. Incidence rate of hospital admissions according to the main ICD-10 chapters.** **Comparison between individuals with and without diabetes, excluding prevalent diseases for each respective ICD-chapters.**

| **Hospitalizations according to main ICD-10 chapters**  **(ICD-10 codes)** | **Total number of hosp after exclusion of prevalent hosp** |  | | **Model 1** | | **Model 2** | | |
| --- | --- | --- | --- | --- | --- | --- | --- | --- |
|  |  | **Number of individuals in the total population with**  **≥ 1 hospitalization** | **Median (IQR)** | **IRR**  **(95% CI)** | ***P-value*** | **IRR**  **(95% CI)** | ***P-value*** | ***Bonferroni corrected***  ***p-value*** |
| **All-cause hospitalizations** | 10,127 | 6,652 | 3 (1-5) | 1.52  (1.31-1.77) | **<0.001** | 1.33  (1.15-1.56) | **<0.001** | **0.003** |
| **Infectious diseases**  **(A00–B99)** | 24,726 | 1,563 | 1 (1-1) | 3.09  (2.66– 3.59) | **<0.001** | 2.55  (2.19– 2.98) | **<0.001** | **<0.001** |
| **Cancers**  **(C00–D48)** | 22,122 | 5,421 | 2 (1-3) | 0.97  (0.87– 1.09) | 0.64 | 0.96  (0.86– 1.07) | NS | NS |
| **Haematological diseases**  **(D50–D89)** | 25,479 | 483 | 1 (1-1) | 1.90  (1.41– 2.56) | **<0.001** | 1.66  (1.23– 2.26) | **0.001** | **0.02** |
| **Endocrine diseases**  **(E00–E90)** | 24,813 | 1,106 | 1 (1-2) | 8.35  (7.23– 9.64) | **<0.001** | 6.53  (5.62–7.58) | **<0.001** | **<0.001** |
| **Psychiatric diseases**  **(F00–F99)** | 24,766 | 526 | 1 (1-1) | 1.89  (1.42– 2.51) | **<0.001** | 1.92  (1.43– 2.56) | **<0.001** | **0.001** |
| **Neurological diseases**  **(G00–G99)** | 24,501 | 1,563 | 1 (1-1) | 1.96  (1.65– 2.33) | **<0.001** | 1.81  (1.52– 2.16) | **<0.001** | **<0.001** |
| **Cardiovascular diseases**  **(I00–I99)** | 22,606 | 6,860 | 2 (1-3) | 2.11  (1.92- 2.32) | **<0.001** | 1.76  (1.60– 1.94) | **<0.001** | **<0.001** |
| **Respiratory diseases**  **(J00–J99)** | 24,027 | 3,095 | 1 (1-2) | 1.62  (1.43– 1.83) | **<0.001** | 1.63  (1.43– 1.85) | **<0.001** | **<0.001** |
| **Digestive diseases**  **(K00–K93)** | 21.361 | 4,007 | 1 (1-2) | 1.35  (1.18– 1.54) | **<0.001** | 1.18  (1.03– 1.35) | **0.02** | NS |
| **Dermatological diseases**  **(L00–L99)** | 25,235 | 257 | 1 (1-1) | 4.81  (3.52– 6.58) | **<0.001** | 3.96  (2.87– 5.49) | **<0.001** | **<0.001** |
| **Musculoskeletal diseases**  **(M00–M99)** | 23,510 | 3,697 | 1 (1-2) | 1.27  (1.10– 1.46) | **<0.001** | 1.09  (0.94– 1.26) | 0.25 | NS |
| **Genitourinary diseases**  **(N00–N99)** | 21,920 | 3,260 | 1 (1-2) | 2.13  (1.87– 2.42) | **<0.001** | 1.81  (1.59– 2.07) | **<0.001** | **<0.001** |
| **Unclassified symptoms, (R00–R99)** | 22,537 | 2,822 | 1 (1-1) | 1.91  (1.66– 2.20) | **<0.001** | 1.65  (1.43– 1.90) | **<0.001** | **<0.001** |
| **Injuries and poisoning**  **(S00–T98)** | 22,779 | 528 | 1 (1-1) | 2.93  (2.25– 3.82) | **<0.001** | 2.91  (2.22– 3.81) | **<0.001** | **<0.001** |

*Abbreviations: CI, confidence interval; ICD-10, 10th revision of the International Classification of Diseases; IQR, interquartile ranges; IRR, incidence rate ratio; NS, not significant.*

Negative binomial regression analysis of number of hospitalizations in patients with prevalent diabetes mellitus compared with non-diabetic individuals, stratified according to the main ICD-10 chapters with reported incidence rate ratio.

Model 1: adjusted for age and sex.

Model 2: adjusted for age, sex, apolipoprotein A1, apolipoprotein B, body mass index, current smoking, antihypertensive treatment, systolic blood pressure, prevalent cardiovascular disease, and education level.

**Table S4. Mortality according to the main ICD-10 chapters.** **Comparison between individuals with and without diabetes, excluding prevalent diseases for each respective ICD-chapters.**

| **Deaths according to main ICD-10 chapters**  **(ICD-10 codes)** |  | **Model 1** | | **Model 2** | | |
| --- | --- | --- | --- | --- | --- | --- |
|  | **Number of deaths in total population (%)** | **HR**  **(95% CI)** | ***P-value*** | **HR**  **(95% CI)** | ***P-value*** | ***Bonferroni corrected***  ***p-value*** |
| **All-cause deaths** | 6750 (26.4) | 1.93  (1.76 – 2.10) | **<0.001** | 1.77  (1.62 – 1.93) | **<0.001** | **<0.001** |
| **Infectious diseases**  **(A00–B99)** | 97 (0.4) | 2.64  (1.37 – 5.08) | **0.004** | 2.20  (1.13 – 4.29) | **0.02** | NS |
| **Cancers**  **(C00–D48)** | 2,151 (9.7) | 1.26  (1.05 – 1.53) | **0.02** | 1.25  (1.03 – 1.52) | **0.02** | NS |
| **Haematological diseases**  **(D50–D89)** | 12 (0.00) | 4.63  (1.00 – 21.38) | 0.05 | 2.75  (0.55–13.72) | 0.23 | NS |
| **Endocrine diseases**  **(E00–E90)** | 118 (0.5) | 15.28  (10.45 – 22.36) | **<0.001** | 10.29  (6.89-15.35) | **<0.001** | **<0.001** |
| **Psychiatric diseases**  **(F00–F99)** | 227 (0.9) | 1.41  (0.79 – 2.52) | 0.25 | 1.42  (0.79 – 2.56) | 0.24 | NS |
| **Neurological diseases**  **(G00–G99)** | 252 (1.0) | 1.11  (0.60 – 2.03) | 0.75 | 1.17  (0.64 – 2.16) | 0.61 | NS |
| **Cardiovascular diseases**  **(I00–I99)** | 1,613 (7.1) | 2.40  (2.02 – 2.85) | **<0.001** | 2.05  (1.72 – 2.45) | **<0.001** | **<0.001** |
| **Respiratory diseases**  **(J00–J99)** | 335 (1.4) | 0.96  (0.55 – 1.67) | 0.88 | 1.08  (0.62 – 1.90) | 0.78 | NS |
| **Digestive diseases**  **(K00–K93)** | 139 (0.7) | 1.61  (0.82 – 3.17) | 0.17 | 1.50  (0.75 – 2.29) | 0.25 | NS |
| **Dermatological diseases**  **(L00–L99)** | Only 9 deaths | | | | | |
| **Musculoskeletal diseases**  **(M00–M99)** | 20 (0.1) | 1.32  (0.18 – 9.90) | 0.77 | 1.41  (0.18 – 10.83) | 0.74 | NS |
| **Genitourinary diseases**  **(N00–N99)** | 47 (0.2) | 1.69  (0.52 – 5.45) | 0.38 | 1.64  (0.50 – 5.38) | 0.41 | NS |
| **Unclassified symptoms, (R00–R99)** | 148 (0.7) | 1.63  (0.83 – 3.20) | 0.16 | 1.55  (0.78 – 3.06) | 0.21 | NS |
| **Injuries and poisoning(S00–T98)** | 171 (0.8) | 1.83  (1.04 – 3.23) | **0.04** | 1.86  (1.04 – 3.30) | **0.04** | NS |

*Abbreviations: CI, confidence interval; HR, hazard ratio; ICD-10, 10th revision of the International Classification of Diseases; NS, not significant.*

Cox regression analysis of number of deaths in patients with prevalent diabetes mellitus compared with non-diabetic stratified according to the main ICD-10 chapters with reported hazard ratio.

Model 1: adjusted for age and sex

Model 2: adjusted for age, sex, apolipoprotein A1, apolipoprotein B, current smoking, body mass index, antihypertensive treatment, systolic blood pressure, prevalent cardiovascular disease, and education level.

**Table S5. Incidence rate of hospital admissions according to the main ICD-10 chapters.** **Comparison between individuals with and without prevalent diabetes, excluding individuals with incident diabetes (n=22,381).**

| **Hospitalizations according to main ICD-10 chapters**  **(ICD-10 codes)** |  | | **Model 1** | | **Model 2** | | |
| --- | --- | --- | --- | --- | --- | --- | --- |
|  | **Number of individuals in the total population with**  **≥ 1 hospitalization** | **Median (IQR)** | **IRR**  **(95% CI)** | ***P-value*** | **IRR**  **(95% CI)** | ***P-value*** | ***Bonferroni corrected***  ***p-value*** |
| **All-cause hospitalizations** | 16,223 | 3 (2-6) | 1.66  (1.59-1.75) | **<0.001** | 1.63  (1.54-1.73) | **<0.001** | **<0.001** |
| **Infectious diseases**  **(A00–B99)** | 1,334 | 1 (1-1) | 3.12  (2.72– 3.58) | **<0.001** | 2.77  (2.37– 3.32) | **<0.001** | **<0.001** |
| **Cancers**  **(C00–D48)** | 5,715 | 2 (1-3) | 0.84  (0.73– 0.96) | **0.01** | 0.78  (0.64– 0.95) | **0.01** | NS |
| **Haematological diseases**  **(D50–D89)** | 392 | 1 (1-1) | 1.96  (1.49– 2.58) | **<0.001** | 1.73  (1.26– 2.36) | **<0.001** | **<0.001** |
| **Endocrine diseases**  **(E00–E90)** | 993 | 1 (1-2) | N/A | | | | |
| **Psychiatric diseases**  **(F00–F99)** | 546 | 1 (1-1) | 1.81  (1.45– 2.25) | **<0.001** | 1.96  (1.52– 2.55) | **<0.001** | **0.001** |
| **Neurological diseases**  **(G00–G99)** | 1,412 | 1 (1-2) | 1.86  (1.60– 2.17) | **<0.001** | 1.73  (1.46– 2.06) | **<0.001** | **<0.001** |
| **Cardiovascular diseases**  **(I00–I99)** | 7,151 | 2 (1-3) | 2.37 (2.12- 2.64) | **<0.001** | 1.98  (1.76– 2.24) | **<0.001** | **<0.001** |
| **Respiratory diseases**  **(J00–J99)** | 2,936 | 1 (1-2) | 1.67  (1.39– 2.00) | **<0.001** | 1.78  (1.45– 2.19) | **<0.001** | **<0.001** |
| **Digestive diseases**  **(K00–K93)** | 4,479 | 1 (1-2) | 1.39  (1.21– 1.59) | **<0.001** | 1.35  (1.16– 1.57) | **0.02** | NS |
| **Dermatological diseases**  **(L00–L99)** | 204 | 1 (1-1) | 4.72  (3.53– 6.31) | **<0.001** | 4.01  (2.88– 5.57) | **<0.001** | **<0.001** |
| **Musculoskeletal diseases**  **(M00–M99)** | 3,837 | 1 (1-2) | 1.37  (1.18– 1.59) | **<0.001** | 1.16  (0.98– 1.37) | 0.08 | NS |
| **Genitourinary diseases**  **(N00–N99)** | 3,483 | 1 (1-2) | 2.18  (1.89– 2.51) | **<0.001** | 1.91  (1.64– 2.34) | **<0.001** | **<0.001** |
| **Unclassified symptoms, (R00–R99)** | 2,929 | 1 (1-1) | 2.16  (1.88– 2.48) | **<0.001** | 1.83  (1.57– 2.13) | **<0.001** | **<0.001** |
| **Injuries and poisoning**  **(S00–T98)** | 562 | 1 (1-1) | 2.21  (1.77– 2.77) | **<0.001** | 2.88  (2.24– 3.69) | **<0.001** | **<0.001** |

*Abbreviations: CI, confidence interval; ICD-10, 10th revision of the International Classification of Diseases; IQR, interquartile ranges; IRR, incidence rate ratio; N/A, not applicable; NS, not significant.*

Negative binomial regression analysis of number of hospitalizations in patients with prevalent diabetes mellitus compared with non-diabetic individuals, excluding individuals with incident diabetes, stratified according to the main ICD-10 chapters with reported incidence rate ratio.

Model 1: adjusted for age and sex.

Model 2: adjusted for age, sex, apolipoprotein A1, apolipoprotein B, body mass index, current smoking, antihypertensive treatment, systolic blood pressure, prevalent cardiovascular disease, and education level.

**Table S6. Mortality according to the main ICD-10 chapters.** **Comparison between individuals with and without prevalent diabetes, excluding individuals with incident diabetes (n=22,381).**

| **Deaths according to main ICD-10 chapters**  **(ICD-10 codes)** |  | **Model 1** | | **Model 2** | | |
| --- | --- | --- | --- | --- | --- | --- |
|  | **Number of deaths in total population (%)** | **HR**  **(95% CI)** | ***P-value*** | **HR**  **(95% CI)** | ***P-value*** | ***Bonferroni corrected***  ***p-value*** |
| **All-cause deaths** | 5913 (26.4) | 1.88  (1.72 – 2.05) | **<0.001** | 1.67  (1.52 – 1.83) | **<0.001** | **<0.001** |
| **Infectious diseases**  **(A00–B99)** | 81 (0.4) | 2.69  (1.38 – 5.23) | **0.004** | 2.32  (1.12 – 4.44) | **0.02** | NS |
| **Cancers**  **(C00–D48)** | 2,370 (10.6) | 1.10  (0.92 – 1.32) | 0.23 | 1.04  (0.87 – 1.25) | 0.66 | NS |
| **Haematological diseases**  **(D50–D89)** | 9 (0.00) | 5.37  (1.10 – 26.22) | **0.04** | 1.99  (0.35–11.28) | 0.44 | NS |
| **Endocrine diseases**  **(E00–E90)** | 94 (0.4) | 66.97  (41.02 – 109.32) | **<0.001** | 58.81  (35.16-98.40) | **<0.001** | **<0.001** |
| **Psychiatric diseases**  **(F00–F99)** | 207 (0.9) | 1.30  (0.73 – 2.33) | 0.38 | 1.28  (0.71 – 2.32) | 0.41 | NS |
| **Neurological diseases**  **(G00–G99)** | 257 (1.1) | 0.97  (0.54 – 1.74) | 0.75 | 1.01  (0.56 – 1.81) | 0.98 | NS |
| **Cardiovascular diseases**  **(I00–I99)** | 1,924 (8.6) | 2.49  (2.18 – 2.86) | **<0.001** | 1.92  (1.67 – 2.21) | **<0.001** | **<0.001** |
| **Respiratory diseases**  **(J00–J99)** | 359 (1.6) | 1.22  (0.78 – 1.89) | 0.39 | 1.44  (0.92 – 2.25) | 0.11 | NS |
| **Digestive diseases**  **(K00–K93)** | 166 (0.7) | 1.60  (0.91 – 2.83) | 0.10 | 1.29  (0.72 – 2.31) | 0.40 | NS |
| **Dermatological diseases**  **(L00–L99)** | Only 6 deaths | | | | | |
| **Musculoskeletal diseases**  **(M00–M99)** | 25 (0.1) | 0.85  (0.12 – 6.33) | 0.87 | 1.08  (0.14 – 8.13) | 0.94 | NS |
| **Genitourinary diseases**  **(N00–N99)** | 47 (0.2) | 2.28  (0.90 – 5.79) | 0.38 | 1.98  (0.76– 5.15) | 0.16 | NS |
| **Unclassified symptoms, (R00–R99)** | 147 (0.7) | 1.49  (0.78 – 2.83) | 0.23 | 1.42  (0.74 – 2.74) | 0.30 | NS |
| **Injuries and poisoning(S00–T98)** | 199 (0.9) | 1.58  (0.95 – 2.64) | 0.08 | 1.59  (0.94 – 2.70) | 0.08 | NS |

*Abbreviations: CI, confidence interval; HR, hazard ratio; ICD-10, 10th revision of the International Classification of Diseases; NS, not significant.*

Cox regression analysis of number of deaths in patients with prevalent diabetes mellitus compared with non-diabetic stratified according to the main ICD-10 chapters, excluding individuals with incident diabetes, with reported hazard ratio.

Model 1: adjusted for age and sex

Model 2: adjusted for age, sex, apolipoprotein A1, apolipoprotein B, current smoking, body mass index, antihypertensive treatment, systolic blood pressure, prevalent cardiovascular disease, and education level.
